# Socio-demographic factors related to children’s knowledge about their rights to healthcare services in transitional Albania

**DOI:** 10.3389/fpubh.2024.1391265

**Published:** 2024-12-11

**Authors:** Herion Muja, Suela Vasil, Andis Qendro, Timo Clemens, Dorina Toçi, Ervin Toçi, Helmut Brand, Genc Burazeri

**Affiliations:** ^1^Department of International Health, CAPHRI (Care and Public Health Research Institute), Maastricht University, Maastricht, Netherlands; ^2^Institute of Public Health, Tirana, Albania; ^3^University of Medicine, Tirana, Albania; ^4^“Schools for Health”, A Project of the Swiss Development and Cooperation (SDC) Agency, Tirana, Albania; ^5^Faculty of Natural Sciences, University of Tirana, Tirana, Albania; ^5^School of Dentistry, University of Medicine, Tirana, Albania

**Keywords:** Albania, children, rights to healthcare, schoolchildren, sociodemographic factors

## Abstract

**Background:**

Our aim was to assess the level and socio-demographic correlates of knowledge about rights to healthcare services among children in post-communist Albania in order to inform targeted interventions and policies to promote equitable healthcare access for all children.

**Methods:**

An online survey conducted in Albania in September 2022 included a nationwide representative sample of 7,831 schoolchildren (≈54% girls) aged 12–15 years. A structured and anonymous questionnaire was administered inquiring about children’s knowledge on their rights to healthcare services. Binary logistic regression was used to assess the association of children’s knowledge about their rights to healthcare services with socio-demographic characteristics.

**Results:**

Overall, about 78% of the children had knowledge about their rights to healthcare services. In multivariable adjusted logistic regression models, independent “predictors” of lack of knowledge about rights to healthcare services included male gender (OR = 1.2, 95% CI = 1.1–1.3), younger age (OR = 1.3, 95% CI = 1.1–1.4), pertinence to Roma/Egyptian community (OR = 1.6, 95% CI = 1.1–2.2), and a poor/very poor economic situation (OR = 1.3, 95% CI = 1.0–1.6).

**Conclusion:**

Our findings indicate a significantly lower level of knowledge about rights to healthcare services among children from low socioeconomic families and especially those pertinent to ethnic minorities such as Roma/Egyptian communities, which can result in limited access to essential health services, increased vulnerability to health disparities, and barriers to receiving appropriate care and advocacy for their health and well-being. Seemingly, gender, ethnicity, and economic status are crucial for children’s knowledge of their healthcare rights because these factors shape their access to information, influence their experiences with healthcare systems, and can drive policy and practice to address disparities and ensure equitable access to health services. Health professionals and policymakers in Albania and elsewhere should be aware of the unmet needs for healthcare services due to lack of awareness to navigate the system particularly among disadvantaged population groups.

## 1 Introduction

The right to healthcare refers to the acceptance that every individual has a basic right to receive medical care and treatment, regardless of their economic or social status (1, 2). Access to healthcare services, including preventive services, diagnostic services, and treatment, is considered a fundamental human right (3).

Children have a fundamental right to access healthcare services too, recognized by various international human rights instruments, including the Convention on the Rights of the Child (CRC) (4, 5). Under the CRC, children have the right to the highest standard of health possible, including access to healthcare services, nutritious food, clean water, and sanitation (4, 5). The CRC recognizes the importance of family and community-based healthcare services and encourages governments to provide education and support to parents and caregivers to help them promote the health and wellbeing of their children (4, 5).

Children’s rights to healthcare services have continued to evolve over time, alongside with other societal development and progress that has been a hallmark across most human populations (6). Driven by the United Nations Human Rights Office of the High Commissioner, the concept has been expanded referring to the: *“right of the child to the enjoyment of the highest attainable standard of health”* (6).

According to the World Health Organization (WHO), social determinants of health play a major role in explaining health inequalities both between and within countries worldwide (7). More importantly, from a policy perspective, systematic health differences which are avoidable by reasonable action are considered to be unfair (7). Overall, according to the holistic viewpoint on social determinants of health, the marked health inequities between and within countries are caused by the unequal distribution of wealth which is reflected in different aspects of peoples’ lives (7), including access to healthcare services (7, 8). This certainly includes also differences in the level of knowledge and access to information about children’s rights to healthcare (7, 8). Furthermore, these inequities influence not only physical access to healthcare facilities but also the quality of services received, which often varies depending on socioeconomic status (7, 8). Such disparities underscore the need for policies that prioritize equitable healthcare education and resources, ensuring all children and families are aware of and can exercise their healthcare rights effectively (7, 8). In many cases, families with limited resources or education may lack awareness of their entitlements, preventing them from advocating effectively for their children’s healthcare needs (7, 8). From this perspective, assessment of these gaps in knowledge and access to information is essential for promoting equal opportunities in health outcomes and empowering parents and other caregivers to seek necessary care for their children (7, 8).

Children’s health status and health care needs differ from those of other population groups and primary healthcare staff is often on the frontline to address children’s health conditions and provide information to children and their parents on various health-related services (8). However, besides healthcare staff, the school environment is also important for introducing children with the notions of healthcare rights, which are then expanded as the child progresses through the education system (9). Indeed, teachers and school programs play a crucial role in shaping children’s understanding of their rights, providing age-appropriate information that lays the foundation for informed health choices and self-advocacy as they grow (9). Ideally, both the education and health care system need to be congruent and harmonized in the messages conveyed to children about their healthcare rights. When these systems work in unison, they can create a supportive environment that reinforces children’s understanding of their healthcare rights and fosters a sense of agency in managing their own health from a young age (9).

The level of awareness about healthcare rights is determined by various socio-demographic factors (10–12). However, most of the studies report on patients’ populations and only few among general population samples. Hence, the limited focus on the general population leaves an incomplete picture and creates a knowledge gap about the level of understanding of health rights across diverse sociodemographic groupings pertinent to the general population.

A systematic review confined to individuals aged <18 years has summarized a number of health literacy definitions and models for youth or secondary school students, but not for children under the age of 10 or within a primary school context (13). Notably, no specific information on the rights to healthcare services is available regarding population-representative samples of children.

Hence, the information on the prevalence of knowledge about rights to healthcare among children across different populations across the world remains limited. This lack of data on children’s awareness of their healthcare rights globally hinders the development of targeted educational programs, which are essential for empowering young individuals to understand and advocate for their health needs (4, 5, 8). Existing studies often emphasize adult knowledge of healthcare rights, but children’s understanding remains underexplored. Also, there is limited research evaluating the effectiveness of health rights education programs for children in schools worldwide. Additionally, the healthcare rights knowledge of children from marginalized communities (including ethnic minorities, and children from low socioeconomic backgrounds) is often overlooked.

Especially, the evidence about the level and determinants of rights to healthcare services among Albanian children is very scant. In 1991, Albania emerged from the most isolated socialist regime in Eastern Europe and, since, has been striving for establishing a market-oriented economy.

In this context, our aim was to assess the level of knowledge and socio-demographic correlates of rights to healthcare services among schoolchildren in Albania. Research on children’s rights to healthcare in post-communist Albania is essential to address enduring gaps in access and equity resulting from the previous centralized healthcare system, which often neglected marginalized communities. From this perspective, it is crucial for understanding whether all children, regardless of background, exhibit the same level of knowledge about their rights to healthcare as Albania transitions toward a more inclusive and rights-based health system. We hypothesized a lower level of knowledge about rights to healthcare services among schoolchildren belonging to low socioeconomic groups, because they may face barriers regarding the access to information or may have fewer educational resources and reduced healthcare exposure, which can potentially hinder their awareness of available services.

## 2 Methods

An online cross-sectional study was carried out in September 2022 among schoolchildren in Albania. The study population included a nationwide sample of Albanian schoolchildren belonging to the grades 6–9 (age-group: 12–15 years). The sampling frame consisted of all registered schoolchildren attending grades 6–9 (*N* = 123,998).

The survey was available online to all schoolchildren in grades 6–9 for the whole duration of September 2022. A structured, anonymous and self-administered questionnaire was completed online by schoolchildren who firstly agreed to participate in the survey. Schoolchildren made use of the computer labs available at their respective schools and/or their personal/family devices for completing the online survey.

At the end of September 2022, when the online survey was closed, there were 7,928 children who had completed the questionnaire (6.4% of all registered schoolchildren of this age-group). Of these, 97 questionnaires were either incomplete, or invalid. Hence, the sample included in the analysis consisted of 7,831 schoolchildren attending grades 6–9 (age: 12–15 years). On the whole, distribution of the basic socio-demographic factors (gender, age, place of residence) of participants was similar to the overall number of registered schoolchildren belonging to grades 6–9.

Assessment of knowledge about rights to healthcare services was based on the following question: *“Do you know your rights to healthcare services?”* Potential responses were: “yes” vs. “no.” In addition, children were asked (Table 1) whether they knew where to receive healthcare services; whether they were aware of the existence of healthcare facilities in their respective areas; and whether they had ever visited/consulted a health professional other than complying with the vaccination calendar. Potential responses to each question were: “yes” vs. “no.”

**Table 1:** Knowledge about rights by other dimensions of knowledge and practices regarding healthcare services.

Knowledge about healthcare services	Know their rights (*N* = 5,936)	Do not know their rights (*N* = 1721)	*p* ^b^
Do you know where to receive healthcare services?			
Yes	5,703 (96.5)^a^	1,526 (89.0)	<0.001
No	205 (3.5)	189 (11.0)	
Are you aware of existence of healthcare facilities in your area?			
Yes	5,677 (96.3)	1,012 (59.2)	<0.001
No	218 (3.7)	698 (40.8)	
Excluding vaccination calendar, have you ever consulted/visited a health professional?			
Yes	4,815 (81.9)	776 (45.5)	<0.001
No	1,061 (18.1)	928 (54.5)	

a Absolute numbers and their respective column percentages (in parentheses). Discrepancies in the totals are due to the missing covariate values.

b *p-values from Fisher’s exact test.*

Socio-demographic factors included gender (boys vs. girls), age (in the analysis dichotomized into: 12–13 years vs. 14–15 years), place of residence (urban vs. rural areas), ethnicity (ethnic Albanians vs. Roma/Egyptian communities), maternal education (in the analysis dichotomized into: low education vs. middle/high education), and family economic situation (in the analysis dichotomized into: poor/very poor vs. not poor).

The study was approved by the Albanian Ministry of Education and Sport in June 2022. All schoolchildren were informed by their respective teachers about the aim and procedures of the study and were explained in sufficient detail particularly the aspects related to anonymousness of the survey and the successive aggregated data analysis. Furthermore, passive consent was sought from the parents through teachers in each respective school, similar to the approach employed in all the other school-based studies conducted in Albania including the periodic “Health Behavior in School-Aged Children” surveys^1^. Also, all teachers were carefully instructed to provide correct information to schoolchildren.

Fisher’s exact test was used to compare the distribution of socio-demographic factors (gender, age, place of residence, ethnicity, maternal education and economic situation) between schoolchildren with and without knowledge about their rights to healthcare services (Table 2). Similarly, Fisher’s exact test was employed to compare the distribution of selected dimensions related to knowledge and practices regarding healthcare services (knowledge about places where to receive healthcare services; awareness about the existence of health facilities in the respective living areas; and visits/consultations with health professionals) between children with and without knowledge about their rights to healthcare services (Table 1).

**Table 2:** Knowledge about rights to healthcare services by socio-demographic characteristics in a nationwide sample of Albanian schoolchildren, September 2022.

Socio-demographic factor	Know their rights (*N* = 5,936)	Do not know their rights (*N* = 1721)	*p* ^b^
Gender			
Girls	3,255 (79.0)^a^	863 (21.0)	0.001
Boys	2,681 (75.8)	858 (24.2)	
Age			
12–13 years	2,830 (75.6)	915 (24.4)	<0.001
14–15 years	3,106 (79.4)	806 (20.6)	
Place of residence			
Urban areas	3,687 (77.9)	1,046 (22.1)	0.323
Rural areas	2,226 (76.9)	668 (23.1)	
Ethnicity			
Roma/Egyptian community	116 (68.2)	54 (31.8)	0.004
Ethnic Albanian	5,572 (78.0)	1,568 (22.0)	
Mother’s education			
Middle/High	4,701 (77.3)	1,382 (22.7)	0.411
Low	1,198 (78.3)	332 (21.7)	
Economic situation			
Not poor	5,575 (77.9)	1,585 (22.1)	0.006
Poor/very poor	338 (72.2)	130 (27.8)	

a Absolute numbers and their respective row percentages (in parentheses). Discrepancies in the totals are due to the missing covariate values.

b *p-values from Fisher’s exact test.*

On the other hand, binary logistic regression was employed to assess the association of socio-demographic factors (gender, age, place of residence, ethnicity, maternal education and family economic situation) with knowledge of schoolchildren about their rights to healthcare services (dependent/outcome variable). First (Table 3, left panel), crude (unadjusted) odds ratios (OR: lack of knowledge vs. knowledge about rights to healthcare services), their respective 95% confidence intervals (95% CIs) and *p*-values were calculated for all sociodemographic factors [gender (boys vs. girls), age-group (12–13 years vs. 14–15 years), place of residence (urban vs. rural areas), ethnicity (ethnic Albanians vs. Roma/Egyptian community), maternal education (low education vs. middle/high education), and economic situation (poor/very poor vs. not poor)]. Then, all covariates were entered into the logistic regression models and removed in a backward stepwise elimination procedure if their *p*-value exceeded 0.10. Multivariable-adjusted ORs, their respective 95%CIs and *p*-values were calculated from the final models. Hosmer-Lemeshow test was used to assess the overall goodness-of-fit of the multivariable-adjusted regression models (as a rule of thumb, p-values over 0.20 indicate that the logistic models are suitable, i.e., the models fit well the data); the final model (Table 3, right panel) fitted the criterion.

**Table 3:** Association of knowledge about rights to healthcare services with socio-demographic characteristics of schoolchildren; unadjusted and multivariable-adjusted results from binary logistic regression.

Variable	Left panel: unadjusted models		Right panel: multivariable-adjusted models	
	OR (95% CI)^a^	*p* ^a^	OR (95% CI)^b^	*p* ^b^
Gender				
Girls	1.00 (reference)	0.001	1.00 (reference)	0.004
Boys	1.21 (1.08–1.34)		1.18 (1.05–1.32)	
Age-group				
14–15 years	1.00 (reference)	<0.001	1.00 (reference)	<0.001
12–13 years	1.25 (1.12–1.39)		1.27 (1.14–1.42)	
Place of residence				
Urban areas	1.00 (reference)	0.319		
Rural areas	1.06 (0.95–1.12)			
Ethnicity				
Ethnic Albanian	1.00 (reference)	<0.001	1.00 (reference)	0.010
Roma/Egyptian	1.65 (1.19–2.30)		1.55 (1.11–2.17)	
Mother’s education				
Middle-high	1.00 (reference)	0.393		
Low	0.94 (0.82–1.08)			
Economic situation				
Not poor	1.00 (reference)	0.005	1.00 (reference)	0.029
Poor/very poor	1.35 (1.10–1.67)		1.28 (1.03–1.59)	

a Odds ratios (OR: do not know their rights vs. know their rights to healthcare services), 95% confidence intervals (95%CIs) and *p*-values from crude/unadjusted binary logistic regression models.

b Odds ratios (OR: do not know their rights vs. know their rights to healthcare services), 95% confidence intervals (95%CIs) and *p*-values from multivariable-adjusted binary logistic regression models. All variables presented in the table were entered in a backward stepwise elimination procedure with a *p*-value to exit set at *p* > 0.10. Empty cells represent the variables excluded from the final model.

A *p*-value ≤0.05 was considered as statistically significant in all cases. Statistical Package for Social Sciences (SPSS, version 19.0) was used for all the statistical analyses.

## 3 Results

About 54% of study participants were girls; 38% of participants were from rural areas; 2.3% belonged to Roma and/or Egyptian minorities; 16% reported a low maternal education; and 6% of schoolchildren reported a poor and/or a very poor economic situation (data not shown).

**Figure 1:**
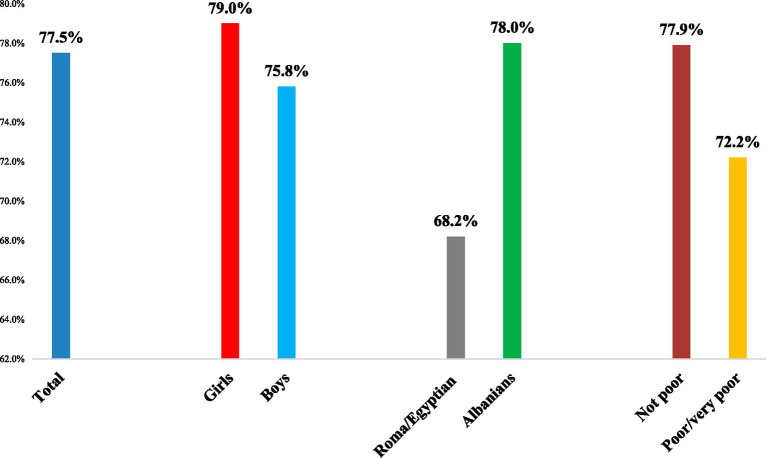
Prevalence of knowledge about rights to healthcare services by selected socio-demographic characteristics in a sample of Albanian schoolchildren, September 2022.

Figure 1 presents the prevalence of knowledge about rights to healthcare by selected sociodemographic characteristics of schoolchildren, whereas Table 2 presents the distribution of knowledge about healthcare rights (“yes” vs. “no”) by all sociodemographic factors measured in this study. Overall, 77.5% of schoolchildren had knowledge about their rights to healthcare. Knowledge about rights to healthcare services was somehow higher among girls than boys (79% vs. about 76%, respectively; *p* < 0.01). Furthermore, it was higher among older schoolchildren (14–15 years) compared with their younger counterparts (about 79% vs. 76%, respectively; *p* < 0.01). In addition, knowledge about rights to healthcare services was considerably higher in the general sample of children (ethnic Albanians) compared with those pertinent to Roma/Egyptian communities (78% vs. 68%, respectively; *p* < 0.01). Also, knowledge about rights to healthcare services was higher in better off children than in those with a poor or very poor economic situation (78% vs. 72%, respectively, *p* = 0.01). Conversely, there were no differences regarding the place of residence (urban vs. rural areas) and/or maternal education.

Table 1 presents the knowledge about rights by other dimensions of knowledge and practices regarding healthcare services. Knowledge about rights to healthcare was higher among children who knew where to receive healthcare services compared to those who did not know where to obtain healthcare services (about 97% vs. 89%, *p* < 0.01). Notably, knowledge about rights to healthcare services was remarkably higher in schoolchildren who were aware of the existence of healthcare facilities in their respective areas compared with children who were not aware of healthcare facilities in their residing areas (96% vs. 59%, respectively; *p* < 0.01). Similarly, knowledge about rights to healthcare services was higher in schoolchildren who had ever visited or consulted a health professional (other than for the purpose of being vaccinated according to the national immunization calendar) compared to those who had never visited/consulted a health professional (82% vs. 46%, respectively; *p* < 0.01).

Table 3 presents the association of knowledge about rights to healthcare services with socio-demographic characteristics of schoolchildren. In crude/unadjusted binary logistic regression models (left panel), lack of knowledge about rights to healthcare services was positively related to male gender (OR = 1.2, 95% CI = 1.1–1.3), younger age (OR = 1.3, 95% CI = 1.1–1.4), belonging to Roma/Egyptian community (OR = 1.7, 95% CI = 1.2–2.3), and a poor/very poor economic situation (OR = 1.4, 95% CI = 1.1–1.7). Thus, the odds of lack of knowledge about rights to healthcare services were 20% higher among males, 30% higher among younger children, 70% higher among children pertinent to Roma/Egyptian families, and 40% higher among those belonging to poor families. Conversely, there was no evidence of associations with place of residence and/or maternal education. Similar findings were evident in multivariable-adjusted binary logistic regression models with all socio-demographic characteristics introduced in a backward stepwise elimination procedure (right panel). Hence, upon multivariable adjustment for all covariates, lack of knowledge about rights to healthcare services was positively associated with male gender (OR = 1.2, 95% CI = 1.1–1.3), younger age (OR = 1.3, 95% CI = 1.1–1.4), pertinence to Roma/Egyptian community (OR = 1.6, 95% CI = 1.1–2.2), and a poor/very poor economic situation (OR = 1.3, 95% CI = 1.0–1.6). Thus, the odds of lack of knowledge about rights to healthcare services were 20% higher among males, 30% higher among younger children, 60% higher among children belonging to Roma/Egyptian families, and 30% higher among those pertinent to poor families.

## 4 Discussion

A main finding of our study consists of the fact that almost 1/4th of schoolchildren that completed the survey (about 23%) did not know their rights to healthcare services. Lack of knowledge to healthcare services was significantly higher among boys, younger children, those belonging to poor families, and especially children pertinent to Roma/Egyptian communities. Hence, our findings highlight significant disparities in the level of knowledge about rights to healthcare among different groupings of Albanian children. Seemingly, the knowledge gap is especially prominent for children pertinent to low socioeconomic backgrounds and ethnic minorities like Roma and Egyptian communities.

Despite the lack of previous studies reporting on children’s level of awareness about their healthcare rights, there are useful insights obtained from research conducted among adults. For example, a large study by Wang et al. (14) involving about four million primary care patients found that women were more likely to consult with primary healthcare doctors compared to men, suggesting that women are more familiar and closer to health and social services and they utilize the healthcare system and preventive services more than men do (15–17). It is possible that such gender difference in the behavior regarding healthcare utilization and consulting might be transferred to children together with gender stereotypes that characterize certain societies including Albania. Indeed, research shows that family and school are the main actors that shape the socialization of children and their affiliation with prevailing social norms and values (18). This might explain why a higher proportion of boys report to be unaware about their healthcare rights in Albania. However, in developing and poor countries, as well as traditional societies, it seems that women underutilize healthcare services more than men (19), due to a mixture of educational, economic, institutional and cultural factors and barriers (19, 20). In this light, transmission of prevailing social norms and values to younger generations seems to be more important in shaping children’s knowledge and attitudes toward healthcare services, including healthcare rights.

In our study, we found that Roma/Egyptian schoolchildren were significantly more likely to be unaware of their healthcare rights compared to prevailing community children. This can be explained by the situation of Roma/Egyptian communities. Research repeatedly has shown that Roma/Egyptian communities are usually disadvantaged, underserved and underpowered in many societies, consistently facing lower education opportunities, higher unemployment rates, stigma, marginalization and racism, and various barriers to access healthcare (21–26). A systematic review has reported that Gipsy and Roma populations across 31 countries in Europe including Albania struggle to exercise their rights to healthcare due to multiple barriers, as well as low literacy levels and experiences of discrimination (21). This comprehensive review included also other post-communist Eastern European countries which share similar patterns with Albania such as Kosovo, North Macedonia, Montenegro, Serbia, Bulgaria and Moldova (21). Our findings are compatible with this systematic review (21), as well as with previous studies conducted in other European countries (22–26), including also former communist countries in Europe (22). Essentially, the worse health profile among Roma and Egyptian minorities is explained by numerous barriers in accessing healthcare (22). Of note, interventions promoting Roma/Egyptian health are often integrated into national policy frameworks, but there is limited evidence of their effectiveness in reaching the marginalized Roma/Egyptian communities (24).

The situation of Roma/Egyptian community in Albania is also difficult (27), which affects Roma/Egyptian children, too (28). In addition, research has shown that low financial status is significantly associated with increased barriers to access healthcare services, through a complex interaction with lack of education, discrimination and distrust in the health system (29). This is a situation where Roma/Egyptian communities are typically found, further explaining why Roma/Egyptian children are more likely of being unaware about their healthcare rights. However, we did not have specific information to conduct a subgroup analysis within the Roma/Egyptian subsample, as “Roma/Egyptian” was coded as a single ethnicity. A subgroup analysis should be conducted in future studies in Albania and elsewhere for identifying specific cultural, economic, and systemic barriers that each of these two subgroup faces (Roma and Egyptian minorities), which hinder their level of knowledge about healthcare rights. Ultimately, this information would be crucial for enabling targeted and effective interventions in each minority subgroup.

Ethiopian mothers from poorest households were about three times more likely to delay early healthcare seeking for their children (30). Conversely, frequent contact with the healthcare system often leads to improved awareness about patient healthcare rights because, among other things, meeting and consulting with the medical staff increases information opportunities and this might include aspects related to patients’ healthcare rights as well (31). This is compatible with our results indicating that, significantly higher proportions of children that are aware of their healthcare rights had ever consulted/visited a health professional compared to children unaware about their healthcare rights. Apparently, the medical encounter enhances information and awareness about health issues in general, including healthcare rights, not only for the parents/adults but also this effect includes children and/or is transmitted to the latter through their parents. In addition, knowledge about healthcare facilities existing in a certain geographical area and knowing where to receive healthcare services, are among the factors that determine healthcare utilization (32).

In our study we did not find evidence of regional disparities when comparing Tirana (the capital, which is the largest and the wealthiest region of the country) with the other regions of Albania. Hence, knowledge about rights to healthcare services was very similar among children pertinent to Tirana and in those from other regions of Albania (78.3% vs. 77.4%, respectively; *p* = 0.55) [data not shown].

The fact that higher proportions of younger children are unaware about their healthcare rights is logical and compatible with stages of development, as well as different healthcare needs and competencies at different ages. Usually, children aged 12–14 years cannot consent to any type of health care, children 14–16 years old can consent to certain simple healthcare procedures without the involvement of parents or carers, and starting from 16 years children can consent to healthcare treatment just as the adults do (33). These stages might be closely linked to the level of awareness about healthcare rights as well.

Our study may have some limitations related to sample representativeness, possibility of information bias, and the study design. Our study included a nationwide sample of schoolchildren aged 12–15 years (grades 6–9). All registered schoolchildren of this age-group were invited to participate over a 1-month period (September 2022). During this time period, 6.4% of schoolchildren completed the online survey which, in absolute terms, constitute a fairly large sample size. More importantly, there were no significant differences regarding the distribution of socio-demographic factors (age, gender and place of residence) between survey participants and the overall number of registered schoolchildren attending grades 6–9. Nevertheless, non-response bias may skew results by underrepresenting disadvantaged groups, potentially masking the true extent of their lack of knowledge on rights to healthcare, ultimately leading to policies that fail to address their specific needs. The instrument of data collection consisted of a previously validated (in a sample of schoolchildren in Tirana), simple and anonymous questionnaire which was completed online by the children at their suitability. Nonetheless, the possibility of information bias cannot be ruled out, as differential reporting between different sociodemographic groupings of children can lead to biased study findings considering variations in cultural norms, literacy, parental involvement, and access to resources which may influence how children understand and respond to survey questions. Also, findings from cross-sectional studies should be interpreted with caution because they capture data at a single point in time, making it difficult to establish causality or account for changes over time.

Despite these possible limitations, our study provides useful and novel evidence about the extent of knowledge about rights to healthcare services and their socio-demographic correlates among Albanian schoolchildren, a transitional country in Southeastern Europe that is striving for ensuring universal health coverage to the overall population.

Our findings point to a significantly lower level of knowledge about rights to healthcare services among Albanian children from low socioeconomic families and especially those pertinent to ethnic minorities such as Roma/Egyptian communities. Health professionals and policymakers in Albania and elsewhere should be aware of the unmet needs for healthcare services due to lack of awareness to navigate the system particularly among disadvantaged population groups. One concrete suggestion for Albania and other similar settings would be to develop culturally tailored outreach programs and educational campaigns to raise the awareness about healthcare rights and services, specifically targeting disadvantaged socioeconomic groups, including ethnic minorities such as Roma and Egyptian communities. Also, policymakers in Albania should consider implementation of community-based health navigation support systems to assist underserved populations in understanding their rights, accessing healthcare services when needed, and navigating effectively the healthcare system.

At an international level, our findings underscore the broader issue of health inequities among marginalized populations, reinforcing the need for global policy frameworks and cross-border collaborations to prioritize rights and access to healthcare services for disadvantaged socioeconomic population categories and ethnic minorities.

Future studies addressing healthcare rights for children should focus on several key gaps to deepen understanding and promote effective advocacy and policy formulation. Existing studies often emphasize adult knowledge of healthcare rights, but children’s understanding remains underexplored. Hence, future studies should investigate the level of awareness and understanding of healthcare rights among children across different age groups, considering age-appropriate methods and tools for measuring children’s comprehension. Also, considering the limited research evaluating the effectiveness of health rights education programs for children in schools, future studies should examine the impact of integrating healthcare rights education into school curricula. More specifically, future research should examine which educational methods (e.g., interactive workshops, digital tools, or the like) are most effective in improving children’s knowledge of their healthcare rights.

## Data Availability

The raw data supporting the conclusions of this article will be made available by the authors, without undue reservation.

## Footnotes

^1^ https://www.who.int/europe/initiatives/health-behaviour-in-school-aged-children-(hbsc)-study
