# Supplementary material for: Socio-demographic factors related to children’s knowledge about their rights to healthcare services in transitional Albania
Source: Front Public Health. 2024 Dec 11;12:1391265. doi: 10.3389/fpubh.2024.1391265 (PMC11668750; doi:10.3389/fpubh.2024.1391265)
Supplement: Supplementary file 1 [file Data_Sheet_1.pdf]

Questionnaire

Do you know your rights to healthcare services?

Yes ☐
No ☐

Do you know where to receive healthcare services?

Yes ☐
No ☐

Are you aware of the existence of healthcare facilities in your living area?

Yes ☐
No ☐

Have you ever visited/consulted a health professional other than complying with the vaccination calendar?

Yes ☐
No ☐

Socio-demographic factors

Gender:

Boy ☐
Girl ☐

Age:

12 years ☐
13 years ☐
14 years ☐
15 years ☐

Place of residence:

Urban area ☐
Rural area ☐

Ethnicity:

Ethnic Albanian ☐
Roma/Egyptian community ☐

Maternal education:

Low ☐
Middle ☐
High ☐

Family economic situation:

Not poor ☐
Poor ☐
Very poor ☐
